# Age‐related differences in the translational landscape of mammalian oocytes

**DOI:** 10.1111/acel.13231

**Published:** 2020-09-20

**Authors:** Edgar del Llano, Tomas Masek, Lenka Gahurova, Martin Pospisek, Marketa Koncicka, Anna Jindrova, Denisa Jansova, Rajan Iyyappan, Kristina Roucova, Alexander W. Bruce, Michal Kubelka, Andrej Susor

**Affiliations:** ^1^ Laboratory of Biochemistry and Molecular Biology of Germ Cells Institute of Animal Physiology and Genetics CAS Libechov Czech Republic; ^2^ Laboratory of RNA Biochemistry Department of Genetics and Microbiology Faculty of Science Charles University in Prague Prague Czech Republic; ^3^ Laboratory of Early Mammalian Developmental Biology (LEMDB) Department of Molecular Biology and Genetics Faculty of Science University of South Bohemia Ceske Budejovice Czech Republic

## Abstract

Increasing maternal age in mammals is associated with poorer oocyte quality, involving higher aneuploidy rates and decreased developmental competence. Prior to resumption of meiosis, fully developed mammalian oocytes become transcriptionally silent until the onset of zygotic genome activation. Therefore, meiotic progression and early embryogenesis are driven largely by translational utilization of previously synthesized mRNAs. We report that genome‐wide translatome profiling reveals considerable numbers of transcripts that are differentially translated in oocytes obtained from aged compared to young females. Additionally, we show that a number of aberrantly translated mRNAs in oocytes from aged females are associated with cell cycle. Indeed, we demonstrate that four specific maternal age‐related transcripts (*Sgk1*,* Castor1*,* Aire* and *Eg5*) with differential translation rates encode factors that are associated with the newly forming meiotic spindle. Moreover, we report substantial defects in chromosome alignment and cytokinesis in the oocytes of young females, in which candidate CASTOR1 and SGK1 protein levels or activity are experimentally altered. Our findings indicate that improper translation of specific proteins at the onset of meiosis contributes to increased chromosome segregation problems associated with female ageing.

## Graphical abstract

Schematic model depicting that maternal age affects the translation of specific mRNAs in oocytes. Fully grown oocytes from different maternal age have similar transcriptomes at the GV stage. However, shortly after NEBD, some of these transcripts are not translated at the same level between YF and AF oocytes, such as *Sgk1*,* Aire*,* Castor1* and *Eg5*. These differences in translation result in different protein expression which in turn lead to chromosome misalignments and cytokinesis abnormalities in AF oocytes.

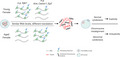

## 1 INTRODUCTION

The quality of oocytes (female germ cells) is an essential factor for successful sexual reproduction. Mammalian oocyte development is a complex and long process beginning during embryogenesis and then arresting in the first meiotic prophase. It is not until puberty that oocytes can finally be recruited for ovulation, by reinitiating meiosis and culminating in eventual fertilization. It is accepted that chromosomal aneuploidy, a consequence of chromosomal segregation errors during meiosis, is one of the most common causes of poor oocyte quality, leading to embryo lethality or severe developmental disabilities (Savva, Walker, & Morris, 2010). Importantly, it has been reported that most chromosomal segregation aberrations take place during the first meiotic division, occurring after meiotic resumption and Nuclear Envelope Break Down (NEBD)(Hassold & Hunt, 2001). Precisely, it is shortly after NEBD when chromosome condensation and assembly take place at the newly forming meiotic spindle (Schuh & Ellenberg, 2007).

Chromosomal aneuploidy in human oocytes is not a rare event, exemplified by a 20% incidence in 32‐year‐old women that further increases with age, reaching 60%–80% in women aged 42 (Capalbo, Hoffmann, Cimadomo, Maria Ubaldi, & Rienzi, 2017; Jones & Lane, 2013; Kuliev, Zlatopolsky, Kirillova, Spivakova, & Cieslak Janzen, 2011). This so called “age‐related aneuploidy” in oocytes has long been a focus in the study of human reproduction, as women in economically advanced societies are trending to delay their pregnancies (Molina‐García et al., 2019). However, we are still far from fully understanding the mechanisms underlying this phenomenon.

In order to provide enhanced insight into the dysfunctional molecular mechanisms behind age‐related aneuploidy, the transcriptomes of oocytes from young and advanced aged females have been compared, using various animal models (Labrecque & Sirard, 2014), of which mouse has proven to be a suitable one (Jones & Lane, 2013b; Sebestova, Danylevska, Dobrucka, Kubelka, & Anger, 2012). These studies have revealed that there are actual significant differences in the transcriptomes of oocytes from aged females, mainly associated with decreased levels of gene transcripts involved in managing oxidative stress, chromatin structure, genome stability and spindle structure. Nonetheless, another crucial feature of oocyte development must be taken into account. Namely, that oocytes proceed through meiotic maturation and to the post‐fertilization zygote stage in the absence of de novo transcription (De La Fuente et al., 2004; Hamatani, Carter, Sharov, & Ko, 2004). Accordingly, growing oocytes that become arrested at prophase I synthesize large amounts of RNA that is stored for future use during the transcriptionally silent period after meiotic resumption (Fulka, First, & Moor, 1998). Precisely, chromosome condensation and segregation take place in an environment where regulation of translation is a paramount gene expression control mechanism. As such, conventional transcriptome analysis, although undoubtedly useful, might not offer a sufficiently accurate picture of oocyte gene expression on the protein level, as the whole transcriptome includes large pools of stored RNAs not undergoing active translation. To overcome this limitation, polysomal fractionation of oocyte samples can be performed in order to isolate those RNAs which are bound to ribosomes and hence are more likely to be translated into proteins with functional roles during specific stages of meiosis. However, mammalian oocytes are a scarce source of material, which is a big limiting factor to such classical fractionation techniques that typically require millions of cells (Mašek, Valášek, & Pospíšek, 2011). Thus, unsurprisingly, only a few experiments of this kind have been reported (Chen et al., 2011; Potireddy, Vassena, Patel, & Latham, 2006; Scantland et al., 2011). For this purpose, we recently developed a Scarce Sample Polysomal‐profiling (SSP‐profiling) method, which allows the obtaining of polysomal RNAs with high reliability from as little as 200 oocytes (Masek et al., 2020).

In this study, we present what to our knowledge is the first thorough analysis of ribosome‐bound mRNAs in mouse oocytes at the post‐NEBD stage within the context of maternal age. Moreover, we validate several candidate mRNAs exhibiting altered translation in the oocytes of more advanced age females and identify novel gene transcripts and proteins related to mammalian oocyte development and aneuploidy.

## 2 RESULTS

### 2.1 Genome‐wide analysis reveals differential translation of cell cycle players in oocytes from aged females

**Figure 1:**
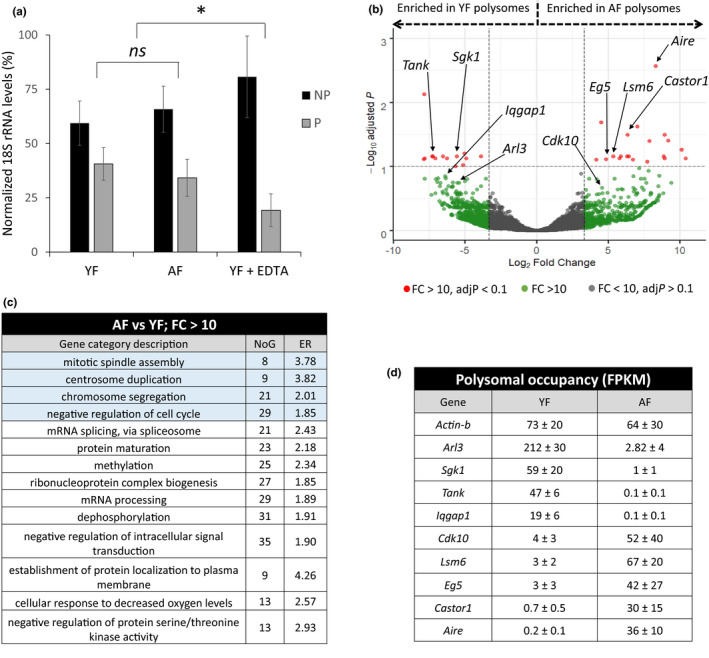
Genome‐wide analysis shows differential translation of cell cycle regulators in oocytes from aged females. (a) qRT‐PCR analysis of the distribution of 18S rRNA between non‐polysomal (NP) and polysomal (P) fractions in oocytes of young female (YF), aged female (AF) mice and in oocytes with disrupted polysomes (YF + EDTA). Data are represented as the mean ± *SEM*, *p < 0.05; *ns*, non‐significant; according to Student's *t* test. (b) Volcano plot displaying gene transcripts differentially enriched in P fractions of oocytes from YF and AF groups; highlighting those with FC > 10 and adjusted‐*p* < 0.1 (red), with only FC > 10 (green) and the rest (grey). Data points with gene names refer to the selected target genes with possible function in female meiosis. See also Table S1. (c) Top enriched ORA categories in differentially enriched genes (FC > 10) from associated polysomal fractions. Categories related to cell division are highlighted in blue. NoG, Number of Genes that belong within a category; ER, Enrichment Ratio. See also Table S2. (d) FPKM values (±standard deviation) for selected target transcripts differentially enriched between YF and AF polysomal fractions that are potentially related to female meiosis. See also Table S1

Most oocyte meiotic errors from aged females occur in meiosis I (Hassold & Hunt, 2001). To test the effect of maternal age on oocyte quality, we performed cold shock treatment on oocytes from young (2 months old, YF) and aged (12 months old, AF) female mice followed by assessment of spindle morphology and chromosome alignment. As expected, after a short time of cold culture, YF oocytes showed organized spindles and chromosomes with abundant signal for kinetochores while most spindles in AF oocytes were partially or totally disassembled (Figure S1). An observation in agreement with the findings of others (Pan, Ma, Zhu, & Schultz, 2008; Sebestova et al., 2012). Taking into account the fact fully grown oocytes are transcriptionally silent and that the transcriptome of GV oocytes does not significantly change with maternal age (Pan et al., 2008), we sought to analyse oocyte translation in relation to maternal age. Interestingly, we have previously analysed global translational rates between different age groups but we did not find any significant differences (Koncicka et al., 2018). Therefore, we hypothesized that the quality decrease in AF oocytes could be due to changes in the translation of specific transcripts rather than altered global translational levels. Consequently, to provide a detailed mechanistic insight into the increased aneuploidy rates of AF oocytes, we compared those polysome‐bound mRNAs of oocytes from young and aged females by Next Generation Sequencing. The translatome comparison was carried out on oocytes which underwent NEBD (3 h after 3‐Isobutyl‐1‐methylxanthine (IBMX) removal); the stage at which chromosomes condense, the new meiotic spindle is forming and transcription is absent. Four individual replicates were used for YF samples and three for AF (200 post‐NEBD oocytes/sample). Using the SSP‐profiling method (Masek et al., 2020) combined with RNA‐Seq, we analysed mRNA content in polysomal and non‐polysomal fractions from oocytes of the two different female groups. Analyses of the YF‐ and AF‐derived fractions did not reveal any significant differences between the two age groups regarding polysomal distribution (Figure 1a). Significant decreases of ribosomal subunits in polysomal fractions were induced by pre‐treatment with Ethylenediaminetetraacetic acid (EDTA), compared to both the YF and AF samples (Figure 1a). As EDTA sequesters magnesium ions to disrupt ribosomal subunit assembly (Scantland et al., 2011), thus reducing polysome levels, this important technical control provides confidence that the RNAs in polysomal fractions of untreated YF and AF oocytes are indeed bound to ribosomes.

Next, for each sample, we pooled the collected fractions into either non‐polysomal (NP, fractions containing free RNAs or RNAs bound to monosomes) or polysomal fractions (P; fractions containing polysome‐bound RNAs). This was followed by ribodepleted RNA‐Seq of NP and P fractions to detect genome‐wide translational differences between the two age groups.

Based on the RNA‐Seq data output, we were able to identify differentially translated mRNAs (Figure 1b). Analysis of P fractions with transcripts with at least bigger than 1 Fragments per Kilobase of exon model per Million reads mapped (FPKM) in one group revealed 3589 (35.06%) mRNAs with more than a five‐fold comparative difference in enrichment between YF and AF oocytes (FC > 5) and 1006 (9.83%) mRNAs with an enrichment fold change above 10 (FC > 10) (Figure 1b and Table S1). From those mRNAs with FC > 10, 623 mRNAs were more abundant in the P fractions of YF oocytes and 383 in P fractions of AF samples. In addition, 37 polysomal differentially enriched mRNAs were statistically significant between the two groups, as calculated by DEseq2 (Love, Huber, & Anders, 2014) (Figure 1b and Table S1). We performed Over‐Representation Analysis (ORA) on the polysomal and highly differentially enriched transcripts exhibiting FC > 10 between the two oocyte groups, thus allowing more meaningful results as they conform a larger gene set (Figure 1c and Table S2). Interestingly, among the significantly enriched categories were those related to cell division, microtubule cytoskeleton (e.g. *Cdk10*,* Cep63*,* Chek2*,* Nde1*,* Spice1* and kinesins: *Ef5 (Kif11)*,* Kif3b*, Kif18a) and to mRNA metabolism (e.g. *Eif3a*,* Prkra*, Rpl38; RNA methyltransferases: *Tfb2m*,* Trmt61a*,* Nsun2*,* Nsun3*), substantiating a possible link between increased aneuploidy in AF oocytes and the transcript‐specific translational alterations. Moreover, those gene transcripts that were differentially enriched between the P fractions of the two oocyte age groups (FC > 10) showed seven hierarchical clusters based on their observed enrichments in non‐polysomal and polysomal fractions (Figure S2 and Table S3). Clusters 2, 5, 6 and 7 appear to comprise gene transcripts with differential translation regulation between the YF and AF groups, as despite having similar abundancies in non‐polysomal (NP) fractions they exhibit opposing enrichment trends in the corresponding polysomal (P) fractions.

In summary, we used genome‐wide analysis to generate a comprehensive dataset of specific mRNAs with differential polysomal abundancy in oocytes from different maternal age‐related oocytes that resumed meiosis.

### 2.2 Specific genes transcripts with different polysomal occupancy between YF and AF oocytes positively correlate with protein expression

To identify candidate genes potentially functionally involved in age‐related aneuploidy, we selected 10 gene transcripts with different polysomal occupancy between YF and AF datasets (Deseq2, *p* < 0.1 and/or FC > 10). We primarily focused on transcripts that might have a potential direct/indirect effect on meiosis, cell division, G2/M‐phase transition or spindle localization according to the current literature (Figure 1b,d). *Actb* mRNA was selected as a control due to similar polysomal occupancy in both oocyte groups.

**Figure 2:**
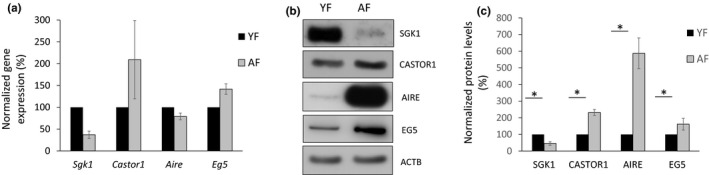
Validation of specific genes with different polysomal mRNA occupancy; positive correlation with protein expression between YF and AF oocytes. (a) ddPCR validation of selected mRNAs in polysomal fractions from YF and AF oocytes (200 oocytes per sample after SSP‐profiling). Values obtained were normalized to ACTB and YF group was set as reference (100%). Data represented as the mean ± *SEM* of three independent experiments. (b) Western Blot analysis of protein abundance of mRNAs coding for selected genes from RNA‐Seq datasets in the two groups of oocytes. ACTB was used as loading control. *N* = 30 oocytes per sample. See also Table S4 for the list of antibodies. (c) Western Blot quantification of protein expression for selected genes. Values obtained were normalized to ACTB and YF group was set as reference (100%). Data are represented as the mean ± *SEM* of at least three independent experiments; *N* = 30 oocytes per sample; *p < 0.05, according to Student's *t* test. See also Table S4 for the list of antibodies

In order to validate the observed differences in polysome association of selected transcripts, each specific mRNA was analysed by Digital Droplet PCR (ddPCR) from newly prepared and pooled YF and AF oocyte polysomal fractions. Due to the inherent scarcity of oocyte samples and the intrinsic difficulty to obtain enough replicates (especially from the limiting numbers of mice and oocytes in the AF group), it was challenging to retrieve flawlessly consistent qPCR results for all assayed transcripts. Nonetheless, we were able to moderately correlate the RNA‐Seq data with the results of ddPCR (Figure 1b,d) for selected candidate mRNAs: Serum/Glucocorticoid Regulated Kinase 1 (*Sgk1*), Cytosolic Arginine Sensor For mTORC1 Subunit 1 *(Castor1)* and Kinesin‐5 protein *(Eg5)* (Figure 2a). In our sequencing datasets, *Sgk1* mRNA was less abundant in the polysomal RNA of AF oocytes, while *Castor1* and *Eg5* mRNA levels were enriched. These relative oocyte age‐related polysome enrichment differences were reflected by ddPCR (Figure 2a). However, despite the robust differences of Autoimmune Regulator (*Aire)* mRNA in polysomal occupancy observed in our RNA‐seq datasets (strongly favouring the AF group), we were not able to detect significant differences by ddPCR between the two groups. Moreover, we did speculate whether the levels of these transcripts would vary not only in the polysomal fractions but throughout the whole transcriptome as a potential consequence of an age‐related differential transcription during the previous oocyte growth period (rather than a differential translation at post‐NEBD stage). We therefore extracted total RNA (without polysomal fractionation) from freshly collected YF and AF post‐NEBD oocytes and assayed the general expression levels of the same transcripts by qRT‐PCR (Figure S3). However, no significant differences in transcript levels were detected, indicating YF and AF oocytes contain similar amounts of *Sgk1*,* Castor1*,* Aire* and *Eg5* transcripts in their total RNA.

Higher polysomal occupancy by specific mRNAs is very likely to correlate with increased synthesis of the corresponding protein. After assaying the differences in *Sgk1*,* Castor1*,* Aire and Eg5* mRNA enrichment in the respective oocyte polysomal RNA pools (Figure 2a), we asked if these differences (as originally identified by RNA‐Seq—Figure 1d) were also reflected at the protein levels. In order to elucidate this, we performed Western blots (WB) from post‐NEBD oocyte samples, from YF and AF groups. We found that SGK1 protein expression positively correlated with (Figure 2b,c) RNA‐seq results (Figure 1b,d). Conversely, the levels of CASTOR1 and EG5 protein, the mRNAs of which were enriched in AF polysomes, were significantly increased in AF‐derived oocytes compared to the YF group (Figure 2b,c). Interestingly, despite not being able to clearly validate different levels in polysome‐associated transcripts of *Aire* by ddPCR, the expression of its protein was greatly increased (up to 10 fold) in the AF group (Figure 2b,c); a result in strong accord with the original RNA‐Seq polysome profiling results (Figure 1d). Control, ACTB expression in oocyte from YF and AF, showed similar level (Figure 2b). Therefore, the validation of significant differences in protein levels of the selected candidate genes correlated positively with those inferred from the RNA‐Seq polysome association analysis. This difference in protein expression between YF and AF, regardless of similar transcript levels in total RNA, indicates that the differential occupation within polysomal fractions for the selected transcripts is directly related to their protein expression.

### 2.3 Selected differentially expressed proteins are localized at the newly forming spindle

**Figure 3:**
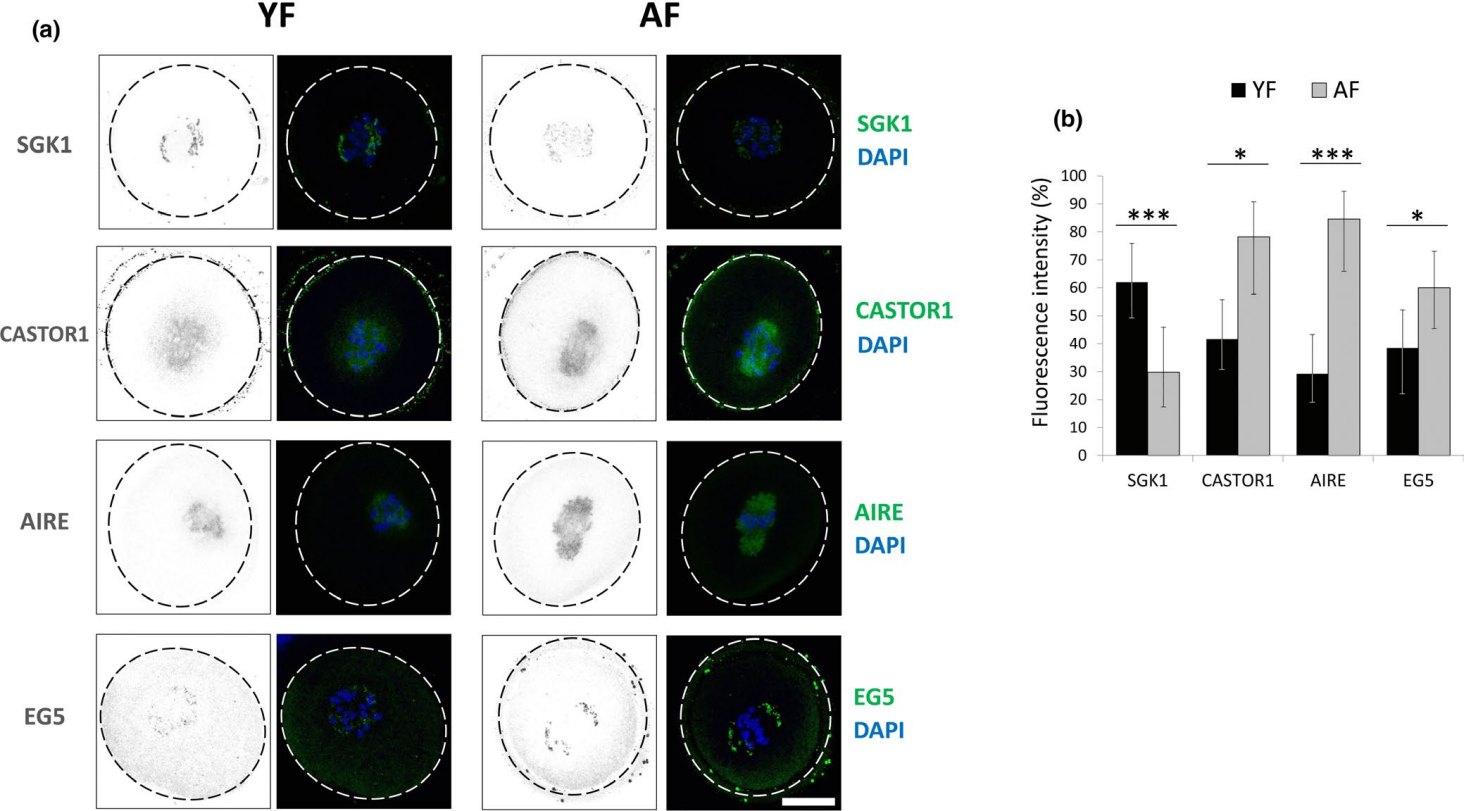
Differentially expressed proteins are localized at newly forming oocyte spindles. (a) Immunocytochemistry shows localization of SGK1, CASTOR1, AIRE and EG5 proteins at newly forming spindles in the post‐NEBD oocytes (YF—left, AF—right). Protein in grey and green scales, DNA (DAPI) in blue. Dashed line indicates oocyte cortex; representative images of at least three independent experiments are shown; scale bar 25 μm. See also Table S4 for the list of antibodies. (b) Immunocytochemistry quantification of protein fluorescence in oocytes from YF (*n* ≥ 48) and AF (*n* ≥ 23) oocytes in at least three independent experiments. Data are presented as the percentage of oocytes with a fluorescence intensity higher than the average in each experiment; *p < 0.05, ***p < 0.001; according to Fisher's exact test, error bars represent 95% confidence intervals by the adjusted Wald method

Most proteins have functional roles within the specific sub‐cellular regions they are localized (Femino, Fay, Fogarty, & Singer, 1998; Jansova, Tetkova, Koncicka, Kubelka, & Susor, 2018). Therefore, to better understand their role in meiosis resumption, we detected localization of selected differentially translated genes from YF and AF oocytes by immunocytochemistry (ICC). Interestingly, we found all four candidate proteins localized in the area of the newly forming spindle but in distinct patterns along or near ß‐tubulin (Figure 3a and Figure S4a). SGK1 and EG5 were found in patches at the multipolar spindle, and CASTOR1 and AIRE were both localized in a similar pattern to newly forming spindle (Figure 3a and Figure S4a). This spindle localization was even more clear after we compared it with Ribosomal Protein S6 (RSP6, a protein with even distribution in the cytoplasm) and when disruption of Tubulin (Nocodazole treatment) changed SGK1 distribution (Figure S4b,c). Moreover, we quantified the fluorescence of individual proteins from ICC and found a positive correlation with our RNA‐Seq (Figure 1b,d) and WB (Figure 2b,c) results. Namely, there was a significant decrease of SGK1 protein fluorescence in AF oocytes compared to YF and significant increase in the protein expression of CASTOR1, AIRE and EG5 (Figure 3b).

Previously, we reported that the timing of meiotic progression of AF‐derived oocytes is faster than in their younger counterparts by about 30 min (Koncicka et al., 2018). Therefore, we asked whether such meiotic progression timing differences may promote translational differences between age groups. We analysed the expression of AIRE and EG5 proteins (by WB and ICC, respectively) in YF oocytes collected 30 min later than the conventional assay time (3 h post IBMX removal) for YF and AF oocytes. We found that additional cultivation did not significantly alter the protein expression to levels previously detected in the AF group (Figure S5). This indicates that the observed translational differences are inherent to translation of selected transcripts in AF oocytes and not due to timing differences.

Collectively, these results confirm the spindle localization of selected candidate proteins and that their differential expression in AF‐derived oocytes (versus YF counterparts) may play a role in spindle assembly, chromosome alignment and cytokinesis.

### 2.4 Perturbation of CASTOR1 and SGK1 protein levels/activity leads to meiotic abnormalities

**Figure 4:**
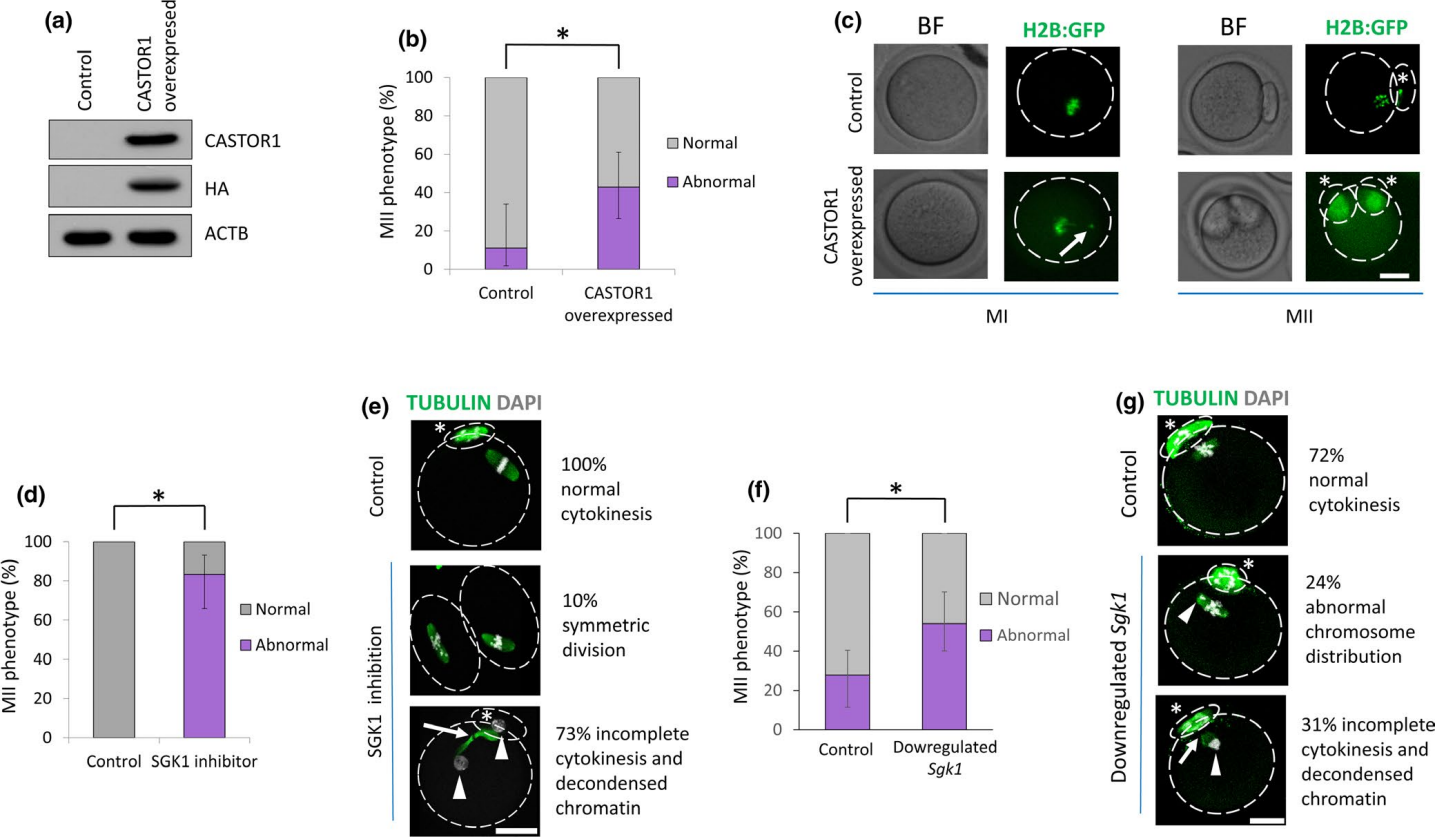
Experimental manipulation of CASTOR1 and SGK1 protein levels/activity causes meiotic abnormalities. (a) Western blot analysis of YF oocytes microinjected with RNA coding for H2B:GFP (Control) or CASTOR1 tagged with HA. ACTB was used as loading control. Representative images from three individual replicates; *N* = 30 oocytes per sample. (b) Quantification of MII oocytes that displayed phenotype with overexpressed CASTOR1 protein from three independent experiments. Data are presented as the percentage of oocytes (*n* ≥ 18) with abnormalities; *p < 0.05; according to Fisher's exact test, error bars represent 95% confidence intervals by the adjusted Wald method. (c) Representative images of chromosome abnormalities in MI and MII oocytes with overexpression of CASTOR1. Bright field (BF, grey) and H2B:GFP (green); dashed line indicates oocyte and polar body cortex; arrow indicates a misaligned chromosome, asterisks denote polar bodies, scale bar 25 μm. (d) Quantification of MII oocytes that displayed phenotype in control (DMSO 0.02%) or SGK1 inhibitor (10 µM) treatment. Data are presented as the percentage of oocytes with abnormalities in control (*n* = 75) and treated (*n* = 75); *p < 0.05; according to Fisher's exact test, error bars represent 95% confidence intervals by the adjusted Wald method. (e) Representative images of oocytes with SGK1 inhibition progressing through cytokinesis. From at least three independent experiments; TUBULIN (green) and DNA (DAPI, grey); dashed line indicates oocyte and polar body cortex; arrow indicates tubulin bridge; arrowheads indicate decondensed chromatin; asterisks denote polar bodies, scale bar 25 μm. (f) Quantification of phenotype of oocytes injected with control RNA (*H2b*:*gfp*) and with down‐regulated *Sgk1* mRNA (*Ds*‐*Sgk1* RNA). Results from two independent experiments; *p < 0.05; according to Fisher's exact test, error bars represent 95% confidence intervals by the adjusted Wald method. See also Figure S7 for validation of down‐regulation of *Sgk1* mRNA. (g) Representative immunocytochemistry images of control oocytes (*H2b*:*gfp* RNA) and *Ds*‐*Sgk1* RNA injected MII oocytes displaying abnormalities. TUBULIN in green and DNA (DAPI) in grey; dashed line indicates oocyte cortex and polar body; arrowheads indicate misalignment and decondensed chromosomes; arrow indicates tubulin bridge; asterisks denote polar bodies, scale bar 25 μm

The sum of the described results indicates that at the post‐NEBD stage, there are a number of mRNAs that exhibit differential polysomal occupancy and consequently altered protein expression levels in AF oocytes compared to YF derived counterparts. We decided to experimentally perturb the protein levels of two selected candidates with opposing differential polysomal occupancy in AF oocytes (CASTOR1 and SGK1, Figures 1b and 3b). As CASTOR1 protein has higher expression levels in AF oocytes, we performed overexpression of the protein in YF oocytes, by microinjection of *Castor1* mRNA (Figure 4a) and consequently analysed their maturation rate and morphology (Figure 4b,c). We found that the rate of progression to the MII stage occurred without major differences in PB extrusion between control (*H2b*:*gfp* mRNA microinjection) and CASTOR1 overexpressed groups (C*astor1*:*Ha* + H2b:*gfp* mRNA microinjection) (61 ± 15% and 61 ± 10%, *n* = 31 and *n* = 18, respectively; *p* < 0.05). However, we did find that 43% of CASTOR1 overexpressing oocytes which reached MII displayed significant abnormalities, namely chromosome misalignment in M‐phase (MI) and/or extrusion of full DNA content into two polar bodies (Figure 4b,c). Although these abnormalities did not affect PB extrusion rates, it is clear that full extrusion of DNA content is deleterious for the further oocyte quality (as well chromosome misalignment would be). On the other hand, translation of *Sgk1* mRNA was significantly decreased in AF oocytes (Figures 1b and 3b), we decided to supress SGK1 protein kinase activity in YF oocytes using the specific SGK1 inhibitor (GSK‐650394; 10 µM). SGK1 affects CDK1 activity (Hiraoka, Hosoda, Chiba, & Kishimoto, 2019), thus we validated successful SGK1 inhibition via decrease of CDK1 (Thr161) phosphorylation in YF oocytes (3 h post IBMX removal in the presence of the inhibitor) (Figure S6). Subsequently, we assayed meiotic progression of YF oocytes treated with vehicle or SGK1 inhibitor. We found that the inhibitor‐treated group had significantly lower numbers of oocytes reaching the MII stage (40 ± 18%; *p* < 0.05; *n* = 75) compared with control vehicle treatment (93 ± 7%; *p* < 0.05; *n* = 75). Moreover, after PB extrusion, 83% of the oocytes treated with SGK1 inhibitor showed two abnormal phenotypes, including symmetric division (10% of MII oocytes) and tubulin non‐disjunction with chromatin decondensation (73% of MII) (Figure 4d,e). Additionally, we also down‐regulated *Sgk1* mRNA (Figure S7) by double‐stranded RNA (Ds‐S*gk1*) microinjection prior to in vitro oocyte maturation. MII oocytes with down‐regulated *Sgk1* mRNA levels had increased frequencies of chromosome misalignment or cytokinetic defects compared to control oocytes (injected with only *H2b*:*gfp* RNA; Figure 4f,g). The effects of D*s*‐*Sgk1* were less pronounced than those obtained after inhibitor treatment, however, with positive correlation.

In summary, by perturbing YF oocytes to mimic the protein expression or activity of CASTOR1 and SGK1 of AF oocytes, we have shown that these proteins have roles involved in meiosis I chromosome alignment and cytokinesis, which are intrinsically impaired in AF‐derived oocytes.

## 3 DISCUSSION

Although several models have been proposed to explain how the age‐associated increase in aneuploidy could occur, further investigation is needed, considering the medical relevance of maternal age‐related aneuploidy. Previous results (Jones & Lane, 2013b; Sebestova et al., 2012) have shown that the mouse is a suitable model system to assess the molecular basis for human age‐associated increase in aneuploidy. A general view is that the quality of the cytoplasm, which includes the transcriptome, is compromised in aged oocytes. However, Pan et al. (2008) have shown that perturbations in the global transcriptome of GV oocytes from females of advanced age are actually minimal.

In this study, we have tackled the old problem of oocyte age‐related aneuploidy from a new perspective. We contribute to this field with a novel genome‐wide analysis of the translation of maternal mRNAs in two different oocyte age groups. While there have been several studies comparing oocyte transcriptomes from young and aged females (Grøndahl et al., 2010; Hamatani, Falco, et al., 2004; Pan et al., 2008), analysis if the actual translatome has until now remained unanalysed. Nonetheless, translation is particularly relevant as the fully grown mammalian oocyte is known to be transcriptionally silent and relies on large amounts of stored RNAs that are utilized when progressing through meiosis. In fact, stored mRNAs can be several times more abundant than those actually undergoing translation at any given point. In order to identify polysome‐bound mRNAs in mouse oocytes, we previously developed a method that enables isolation of very small amounts of polyribosomal bound RNA for a subsequent genome‐wide analysis (Ganesh et al., 2020; Masek et al., 2020). To date, there have been very few experiments applying polysome profiling techniques to oocytes (Chen et al., 2011; Luong, Daldello, Rajkovic, Yang, & Conti, 2020; Potireddy et al., 2006; Scantland et al., 2011), and to our knowledge, we are the first to use it to interrogate the whole translatome in oocytes of different maternal ages.

Research in this study was performed using oocytes at the post‐NEBD stage. This specific meiotic time‐point was deliberately selected, since the process of first meiotic division is being initiated: the meiotic arrest at prophase I is released, chromosomes have recently condensed, the new spindle has begun to assemble (Schuh & Ellenberg, 2007) and translational reprogramming is occurring (Jansova et al., 2018; Susor et al., 2015). Additionally and importantly, chromosomal mis‐segregation is reported to preferentially occur during the first meiotic division. Therefore, uncovering translational differences at this stage between YF and AF oocytes harbours a high potential to better understand age‐related aneuploidy (Ottolini et al., 2015). Earlier studies dealing with whole oocyte transcriptome analysis in correlation with maternal age have focused mainly on the GV and/or MII stages as they represent the two meiotic arrest points. For example, Pan et al. (2008) performed microarray analysis in order to obtain the whole transcriptome of oocytes from young and aged mice females at the GV and MII stages, and reported very little differences in the transcriptome between the age groups at the GV stage (5% with FC > 2) while the transcriptome of MII stage oocytes differed more significantly and correlated with maternal age (33% with FC > 2). A similar trend was observed by Reyes et al. (2017) in a transcriptome study comparing human GV and MII oocytes. However, the transcriptome at the post‐NEBD stage remains unstudied, possibly because whole transcriptome analysis would reveal few differences between the oocytes from different maternal ages, as was previously reported in GV oocytes (Pan et al., 2008). Here, with the advantage of being able to reliably assay polysome‐bound mRNAs, we have been able to detect mRNAs that are actively translated. Thus, we have begun to unveil how the specific oocyte translatome changes with maternal age and how it affects the resulting proteome in the critical developmental window directly after meiotic resumption. For example, the translatome mRNA profiling of post‐NEBD YF and AF oocytes has revealed differentially enriched and relevant ontology categories of mRNA transcripts related to cytokinesis and cell division processes. These ontological categories include specific transcripts with important roles in meiosis (e.g. *Cdk10*,* Cep63*,* Chek2*,* Nde1*,* Spice1* and various kinesins).

Derived from our polysome‐associated mRNA‐Seq screen, we have identified and validated four candidate transcripts that have significantly different polysome enrichment levels between YF‐ and AF‐derived post‐NEBD oocytes: *Sgk1*,* Castor1*,* Aire* and *Eg5*. Intriguingly, the respective proteins of all transcripts localize to the newly forming spindle, which is essential for chromosome alignment and cytokinesis. EG5 is involved in spindle bi‐polarity establishment, microtubule sliding and has been thoroughly studied in mitosis and meiosis (Mann & Wadsworth, 2019). Despite previous whole transcriptome studies failing to report overt differences in *Eg5* mRNA expression between YF and AF oocytes (Grøndahl et al., 2010; Hamatani, Falco, et al., 2004; Pan et al., 2008; Reyes et al., 2017), our SSP‐Profiling approach demonstrates a significant increase in *Eg5* transcript in the polysomes of AF oocytes that then translates to corresponding increased protein expression levels. Interestingly, Liu et al., (2010) and Castillo, Morse, Godfrey, Naeem, and Justice (2007) have shown in other tissue models that overexpression of EG5 can induce aneuploidy and tumorigenesis. Therefore, alteration of EG5 levels in oocytes retains the potential to influence the spindle assembly process, leading to aberrant meiotic progression with abnormal chromosome segregation (Castillo et al., 2007; Kovacovicova, Awadova, Mikel, & Anger, 2016; Liu et al., 2010).

AIRE is another interesting candidate with a possible role in age‐related aneuploidy in mice. Although it has been mainly studied for its role as a transcription factor in immune tolerance control (Björses, Aaltonen, Horelli‐Kuitunen, Yaspo, & Peltonen, 1998), it is known *Aire*‐deficient female mice display infertility albeit without a direct study of oocyte quality (Jasti et al., 2012). Furthermore, an interaction between AIRE and spindle‐associated proteins has been reported to be essential for mitotic spindle assembly in stem cells. In the same study, overexpression of a truncated version of AIRE leads to defective spindles in stem cells (Gu, Lambert, Cockburn, Gingras, & Rossant, 2017). Here, we also find AIRE is localized at the newly forming oocyte meiotic spindle and that *Aire* mRNA is highly translated in aged mice post‐NEBD oocytes compared to their young counterpart. Due to AIRE's main studied role as a transcription factor, the presence of AIRE protein in transcriptionally silent oocytes is not entirely clear. However, as our data demonstrate further research could explain its function in spindle assembly and cytokinesis, with particular emphasis on oocytes from aged females.

The third protein we have identified and validated as up‐regulated in the AF oocytes is CASTOR1. In the presence of arginine, CASTOR1 binds to this amino acid, which prevents its inhibitory effect on GATOR2, ultimately allowing mTOR1 activation (Saxton, Chantranupong, Knockenhauer, Schwartz, & Sabatini, 2016). CASTOR1 localization to the spindle might be connected to mTOR1, which also accumulates to the spindle and its inhibition/down‐regulation leads to meiotic defects (Guo et al., 2018; Susor et al., 2015). Therefore, it is possible that localization of mTOR1 to the assembling spindle, under the control of a strong upstream regulator such as CASTOR1, might be important for its temporal and/or spatial regulation. Indeed, when we experimentally increased CASTOR1 levels in YF oocytes, in order to mimic the increased protein expression as detected in AF oocytes, the most frequently observed anomaly was chromosomal misalignment but also cytokinesis errors, resulting in oocytes devoid of DNA. We note, however, that the increased CASTOR1 levels are several fold higher than those seen in AF oocytes, because it is technically very difficult to achieve levels closer to those seen in AF oocytes. While the latter phenotype is obviously incompatible with further development, chromosome misalignment may lead to aneuploidy and is frequently observed in oocytes from aged females, both mouse and human (Liu & Keefe, 2008; Van den Berg et al., 2011erg et al., 2011; Sebestova et al., 2012). Taken together, these results reveal CASTOR1 as a possible factor relevant to age‐related chromosomal aneuploidy in oocytes.

In contrast to CASTOR1, SGK1 protein expression is lower in AF oocytes compared to YF oocytes. SGK1 is known to regulate the activity of ion channels, solute carriers, enzymes and transcription factors (Lang, Stournaras, Zacharopoulou, Voelkl, & Alesutan, 2018). SGK1 protein kinase activity can be triggered by insulin, follicle stimulation hormone (FSH), corticosterone and thrombin, and is mediated by several upstream kinases including PI3K, PDK1 and mTORC2 (Kobayashi & Cohen, 1999; Pearce, Sommer, Sakamoto, Wullschleger, & Alessi, 2011). Surprisingly, despite its previously described localization in the plasma membrane, we observed it in the region of the post‐NEBD forming oocyte spindle. Paralleling our results, SGK1 levels have also been reported to decline with human ageing (Harries et al., 2012). Moreover, we found that inhibition of SGK1 activity and down‐regulation of *Sgk1* mRNA in YF oocytes visibly leads to cytokinetic errors. Such errors are common in AF‐derived oocytes as evidenced by microtubule defects (Nakagawa & FitzHarris, 2017; Volarcik et al., 1998; Yun, Lane, & Jones, 2014).

**Figure 5:**
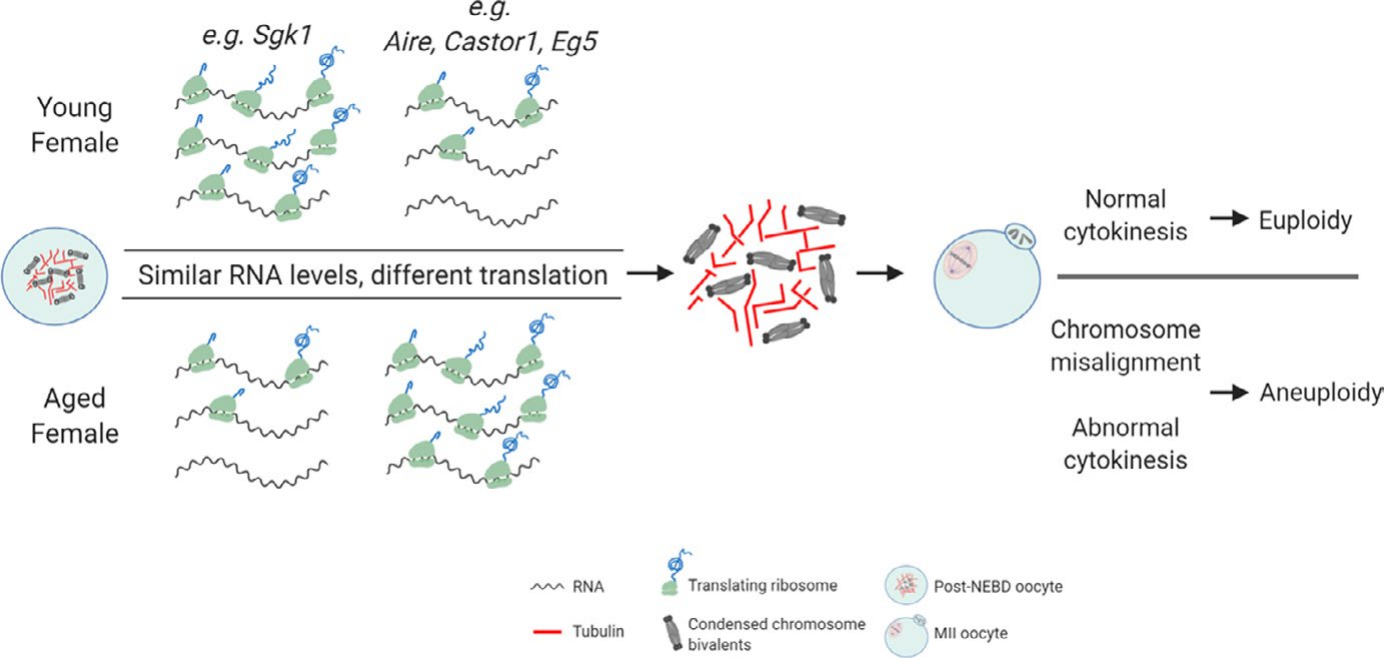
Schematic model depicting maternal age effects on the translation of specific mRNAs in oocytes. Fully grown oocytes from different maternal age have similar transcriptomes at the GV stage. However, shortly after NEBD, some of these transcripts are not translated at the same level between YF and AF oocytes, such as *Sgk1*,* Aire*,* Castor1* and *Eg5*. These differences in translation result in different protein expression which in turn lead to chromosome misalignments and cytokinesis abnormalities in AF oocytes

Altogether, our study has generated a genome‐wide database of mRNA transcripts that occupy post‐NEBD oocyte polysomes in both young and aged mouse oocytes. Furthermore, we have demonstrated the existence of differential polysomal mRNA occupancy in young and aged oocytes, suggesting a different translational program in post‐NEBD oocytes that is dependent on maternal age and correlates with reduced quality of aged oocytes (see scheme in Figure 5); a characteristic phenomenon of both mice and humans. Therefore, our data provide new information related to age‐related translatome/proteome perturbation (including verified candidate gene mRNA/proteins) at the onset of meiosis I, concomitant with spindle formation and resulting cytokinesis.

## 4 MATERIAL AND METHODS

### 4.1 Oocyte collection and culture

GV stage oocytes were collected from ICR mice (bred in‐house) at the age of 2 months old (YF, Young Females group) and 12 months old (AF, Aged Females group) old. Mice were injected 46 h prior to oocyte collection with 5 IU pregnant mare serum gonadothropin (PMSG, HOR 272, ProSpec, Rehovot, Israel). Collection from the ovaries was done in the presence of transfer media (Tetkova and Hancova, 2016) supplemented with 100 µM 3‐isobutyl 1 methylxanthine (IBMX, Sigma‐Aldrich, Darmstadt, Germany) to block meiosis resumption. Fully grown oocytes were selected, isolated and denuded by pipetting followed by culture in M16 media (Sigma‐Aldrich, Darmstadt, Germany) without IBMX at 37°C, 5% CO_2_ for 3 h. Oocytes, that underwent NEBD, were collected for experiments. For obtaining MII oocytes, they were left in culture for additional 13–16 h. For SSP‐profiling, after the 3 h of culture, cycloheximide was added to the M16 media with oocytes at a final concentration of 0.1 mg/ml for 10 min. Oocytes in groups of 200 were transferred to low‐binding retention tubes and frozen at −80°C. In the case of SGK1 inhibition experiments, after IBMX removal GV oocytes were transferred to M16 media supplemented with 0,02% Dimethyl Sulphoxide (DMSO) for solvent vehicle control or 0.02% DMSO + 10 µM GSK‐650394 (Merck, Darmstadt, Germany) for SGK1 inhibitor treatment. All animal work was conducted according to Act No 246/1992 for the protection of animals against cruelty; from 25.09.2014 number CZ02389, issued by Ministry of Agriculture.

### 4.2 Cell lysis and SSP‐profiling

All steps for this protocol were detailed in Masek et al. (2020). Briefly, 200 oocytes per sample were collected in the presence of cycloheximide. Oocytes were lysed by adding 350 µl of lysis buffer and Zirconia‐Silica beads followed by shaking at 30 shakes/second for 1 min and 1 min on ice. This step was repeated three times. Oocyte lysates were centrifuged at 10,000 *g* for 5 min before being then applied to 10%–50% sucrose gradients prepared in SW55Ti ultracentrifuge rotor (Beckman Coulter, Indianapolis, IN, USA). Samples loaded onto sucrose gradients were then centrifuged at 246,000 *g* for 63 min at 4°C. Polysome profiles were collected by pumping 60% sucrose into SW55Ti tubes at a speed of 1.8 ml/min using NE‐1000 syringe pump (New Era Pump Systems, Inc., Farmingdale, NY, USA). Ten equal‐volume fractions, each of 0.5 ml. Absorbance monitoring at 280 nm was performed using an ISCO UA‐5 detector and ISCO UV absorbance reader (Teledyne, ISCO, Lincoln, NE, USA) and profiles were recorded with Clarity Lite software. HEK‐293 cell sample (8 OD_260 nm_) was included in each centrifugation run to monitor quality of separation and one technical negative control containing 200 NEBD + EDTA (100 mM), to sequester magnesium ions and disrupt ribosomal subunits were added. Finally, polysome profiles of all oocyte samples and HEK‐293 technical controls for fractionation were visualized by detecting 18S rRNA levels in collected fractions by qRT‐PCR according to Masek et al. (2020).

### 4.3 Library preparation, sequencing and bioinfomatic analysis

From each sample, fractions were pooled together to create two groups: non‐polysomal (NP) and polysomal (P) fractions. These fractions were concentrated by Clean & Concentrator‐5 (Zymo Research, Irvine, CA, USA) and rRNA depleted by Ribozero‐Gold (Illumina, San Diego, CA, USA). cDNA was produced and amplified with REPLI‐g WTA Single Cell Kit (Qiagen, Hilden, Germany). Afterwards, the amplified cDNA was used to produce libraries using the Nextera DNA Library Prep Kit (Illumina, San Diego, CA, USA) and sequencing performed in Centro Nacional de Analisys Genomico facility (CNAG, Barcelona, Spain) by HiSeq 2500 (Illumina). Sequenced reads were trimmed by Trim Galore! v0.4.1 and mapped to the mouse GRCm38 genome assembly using Hisat2 v2.0.5. Quantification of gene expression was done by fragments per kilobase per million (FPKM) values in Seqmonk v1.40.0. The FC cuff‐off of differentially expressed genes was calculated from FPKM > 1 values (ether in YF or AF) as quantified by Seqmonk v1.40.0 and FC > 10 and FC > 5. Hierarchical clustering was performed and a heatmap was generated using differentially abundant genes (FC > 10) using hierarchical clustering function within Seqmonk v1.40.0. Differentially expressed genes were identified using a DESeq2 package (Love et al., 2014). For gene ontology analysis, we uploaded our datasets into Webgestalt (Liao, Wang, Jaehnig, Shi, & Zhang, 2019). ORA analysis was performed on the 1006 genes which displayed FC > 10 and FPKM > 1 between polysomal fractions of YF and AF. The protocol was described in Masek et al. (2020) and YF samples served to prove validity of the method.

### 4.4 Digital Droplet PCR (ddPCR)

Primer sequences of *Sgk1*,* Castor1*,* Aire* and *Eg5* are listed in Table S4. ddPCR reaction was performed in the QX200™Droplet Reader machine (Bio‐Rad, Hercules, CA, USA) using the QX200™ddPCR™EvaGreen^®^Supermix (Bio‐Rad, Hercules, CA, USA). Absolute quantification was calculated using QuantaSoft™Software (Bio‐Rad, Hercules, CA, USA).

### 4.5 Western blot

Samples of 20–35 oocytes were lysed in 10 µl of 1xReducing SDS Loading Buffer (lithium dodecyl sulphate sample buffer NP 0007 and reduction buffer NP 0004 [Thermo Fisher Scientific, Waltham, MA, USA]) and heated at 100°C for 5 minutes. Proteins were separated in gradient precast 4–12% SDS–PAGE gels (NP 0323, Thermo Fisher Scientific) and transferred onto Immobilon P membrane (IPVD 00010, Millipore, Merck group, Darmstadt, Germany) using a semidry blotting system (Biometra GmbH, Analytik Jena, Jena, Germany) for 25 min at 5 mAxcm^−2^. Blocking was done by 5% skimmed milk dissolved in 0.05% Tween‐Tris buffer saline (TTBS) with pH 7.4 for 1 h. Then, membranes were washed shortly with TTBS and incubated with 1% milk/TTBS diluted primary antibodies (listed in Table S4 and showed target proteins, Figure S8) at 4°C O/N. Secondary antibodies, Peroxidase Anti‐Rabbit Donkey and Peroxidase Anti‐Mouse Donkey (711‐035‐152 and 715‐035‐151, Jackson ImmunoResearch, West Grove, PA, USA) were diluted 1:7500 in 1% milk/TTBS. The membranes were incubated with secondary antibodies for 1 h at room temperature. Protein visualization was performed using chemiluminescent detection ECL, (Amersham, GE Healthcare Life Sciences, Barcelona, Spain) and X‐ray autoradiography. Finally, the films were scanned by a GS‐800 calibrated densitometer (Bio‐Rad Laboratories, CA, USA) and acquired signals were quantified using ImageJ (http://rsbweb.nih.gov/ij/).

### 4.6 Immunocytochemistry

Oocytes were transferred from cultivation media to 4% paraformaldehyde (PFA, Alfa Aesar, Thermo Fisher Scientific, Waltham, MA, USA) in PBS/PVA and left for 15 min followed by permeabilization in 0.1% Triton (X‐100, Sigma‐Aldrich) PBS/PVA for 10 min. Fixed oocytes were then washed in PBS/PVA and incubated with primary antibodies (Table S4) at 4°C O/N. The next day, oocytes were twice washed in PBS/PVA and then incubated with the corresponding secondary antibody that was conjugated with Alexa Fluor 488/594/647 (Invitrogen, Carlsbad, CA, USA) for 1 h at room temperature protected from light. Afterwards, oocytes were washed twice in PBS/PVA and mounted on a glass slide using ProLong™ Gold antifade reagent with DAPI (Invitrogen, Carlsbad, CA, USA). For the cold shock tubulin stability assay, oocytes were cultured for 6.5 h and subsequently incubated for 20 min at 4°C. Afterwards oocytes were fixed and processed for immunocytochemistry. For depolymerization of tubulin, oocytes were treated by 1 µg/ml for three hours. Images of samples were taken with Leica SP5 inverted confocal microscope (Leica Microsystems, Wetzlar, Germany). Images were assembled in software LAS X (Leica Microsystems) and signal intensity from spindle area was quantified with ImageJ. Afterwards, the mean fluorescence intensity of each experiment was calculated. Finally, the fluorescence intensity of each sample was categorized as lower as or higher than the mean.

### 4.7 RNA synthesis, microinjection and live‐cell imaging

*Castor1* and *H2b*:*gfp* RNA were in vitro transcribed (IVT) using corresponding source plasmids as templates (*Castor1*: Chantranupong et al. (2016); *H2b*:*gfp*: provided by Dr. Martin Anger, Laboratory of Cell Division Control, IAPG CAS) and mMESSAGE mMACHINE™ Transcription Kit (Invitrogen, Carlsbad, CA, USA). IVT RNA was injected in GV oocytes at a final concentration of 50 ng/µl in the presence of transfer media with IBMX. Microinjection of GV oocytes was performed using FemtoJet (Eppendorf) and TransferMan NK2 (Eppendorf, Hamburg, Germany) on an inverted microscope Leica DMI 6000B (Leica Microsystems, Wetzlar, Germany). GV oocytes were then placed for 2 h, still in the presence of IBMX, at 37°C, 5% CO_2_. Afterwards, oocytes were removed from IBMX and placed into 4‐well culture chamber (Sarstedt, Prague, Czech Republic) in 15 µl of M16 media covered with mineral oil (M8410; Sigma‐Aldrich) for further cultivation in the inverted microscope for live‐cell monitoring under the same cultivation conditions (Tempcontroller 2000–2 Pecon, and a CO_2_ controller, Pecon, Erbach, Germany). Time lapse images were taken using LAS X software (Leica microsystems, Wetzlar, Germany) every 10 min. Chromosome movement was also assessed by H2B:GFP marker.

For double‐stranded RNA interference experiments, synthesis and annealing of RNA strands were performed as described in Svoboda, (2009). *Ds*‐*sgk1* RNA was injected in 1 µg/µl concentration to GV oocytes with 50 ng/µl of *H2b*:*gfp* RNA. Control oocytes were injected with only *H2b*:*gfp* RNA. Next, oocytes were cultured in presence of IBMX overnight. The next day, a subset of oocytes were analysed for *Sgk1* mRNA expression by qPCR and the remainder matured for 12 h to analyse phenotype.

### 4.8 Statistical analysis

The tools used to determine if the differences between groups were statistically significant were either the Student's *t* test on the averages and *SEM* or Fisher exact test with a 95% confidence interval using adjusted Wald method; *p < 0.05 considered as statistically significant (labelled with a star), ***p < 0.005 was considered as highly statistically significant (labelled with three stars).

## CONFLICT OF INTEREST

The authors certify that they have NO conflict of interest of any kind in the subject matter or materials discussed in this manuscript/article.

## AUTHOR CONTRIBUTIONS

A.S. and E.L. designed the experiments and planned the project; M.K., T.M. and M.P. also contributed intellectually during the development of the project. E.L. was involved in all experiments and performed most of them. T.M. designed, performed and helped in all polysomal fractionation experiments. A.J. and D.J. performed live‐cell imaging experiments, M.K. carried out microinjection experiments, R.Y. performed IVT. L.G. performed and helped in producing sequencing libraries and carried out bioinformatics analysis. M.P. and K.R. also contributed to bioinformatics analysis. E.L. and A.S. wrote the manuscript, all authors edited manuscript, A.S. conceived and supervised the project.

## Data Availability

RNA‐Seq data regarding YF and AF oocytes are deposited in Gene Expression Omnibus database. AF data accession number: PENDING. YF accession number, as stated in (Masek et al., 2020): GSE121358.
