# Supplementary material for: Age‐related differences in the translational landscape of mammalian oocytes
Source: Aging Cell. 2020 Sep 20;19(10):e13231. doi: 10.1111/acel.13231 (PMC7576272; doi:10.1111/acel.13231)
Supplement: Supplementary file 1 [file ACEL-19-e13231-s001.pdf]

Supplementary Fig. 1

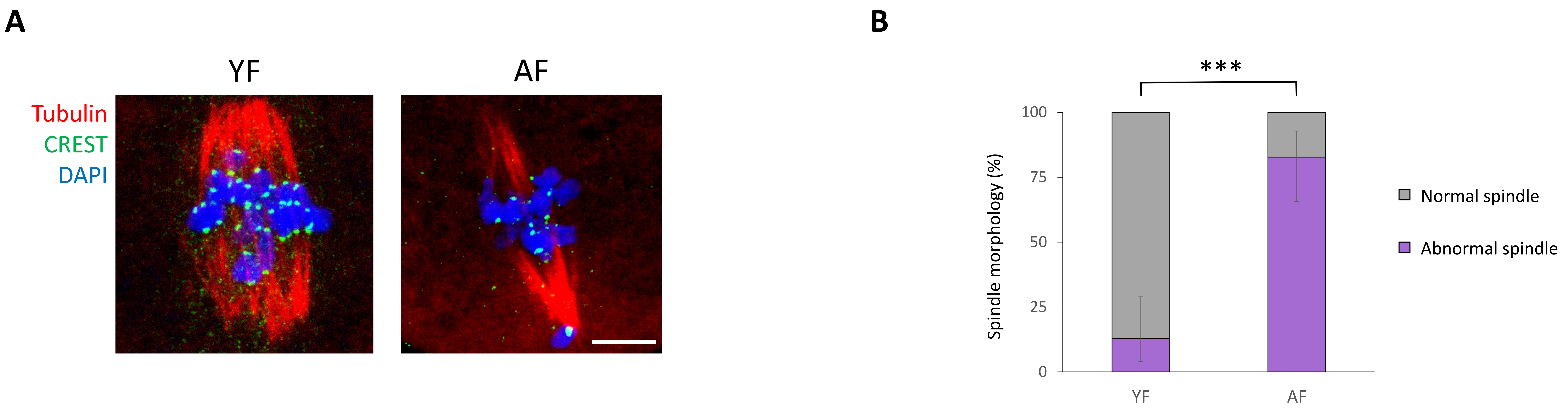

Supplementary Fig. 2

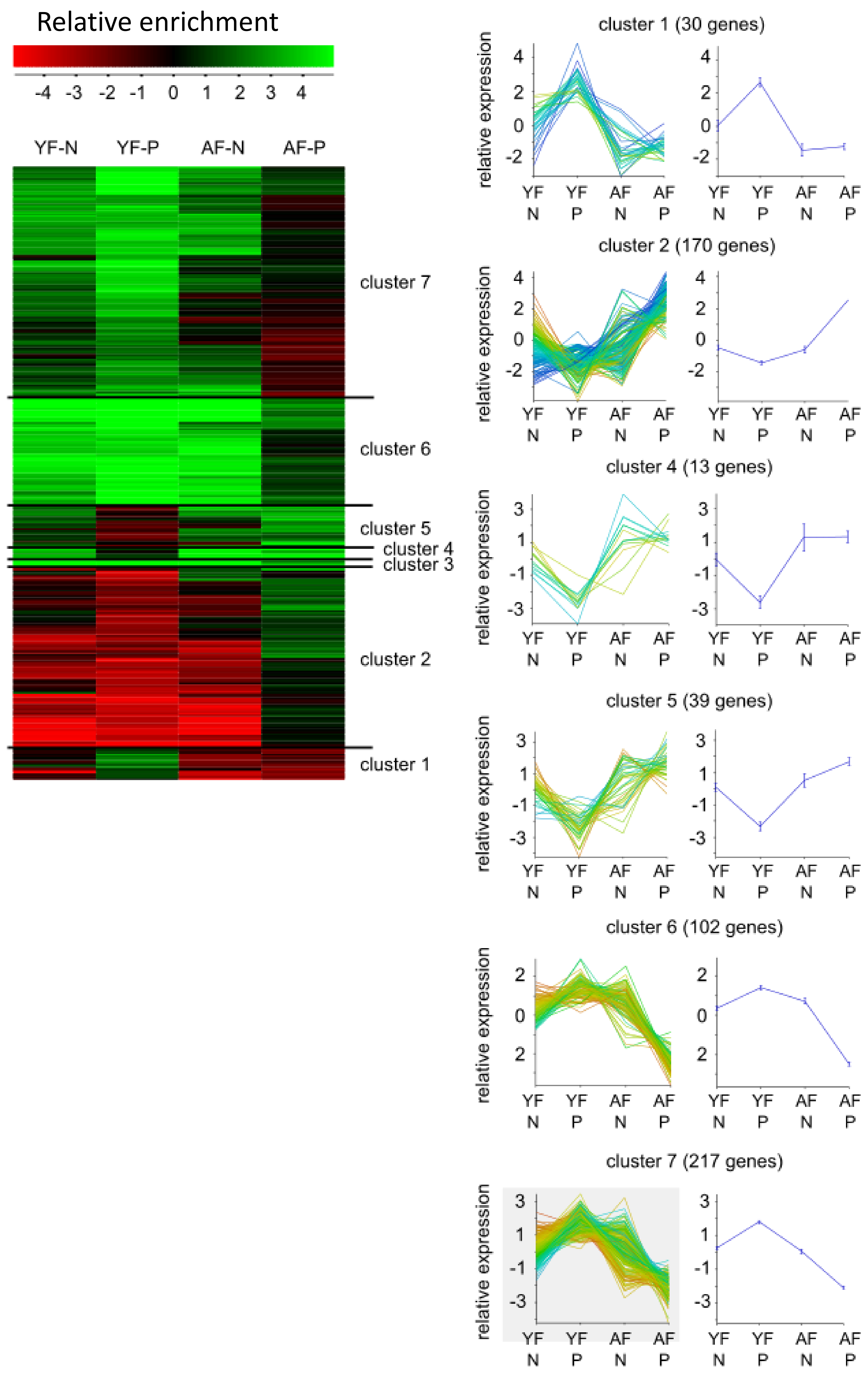

Supplementary Fig. 3

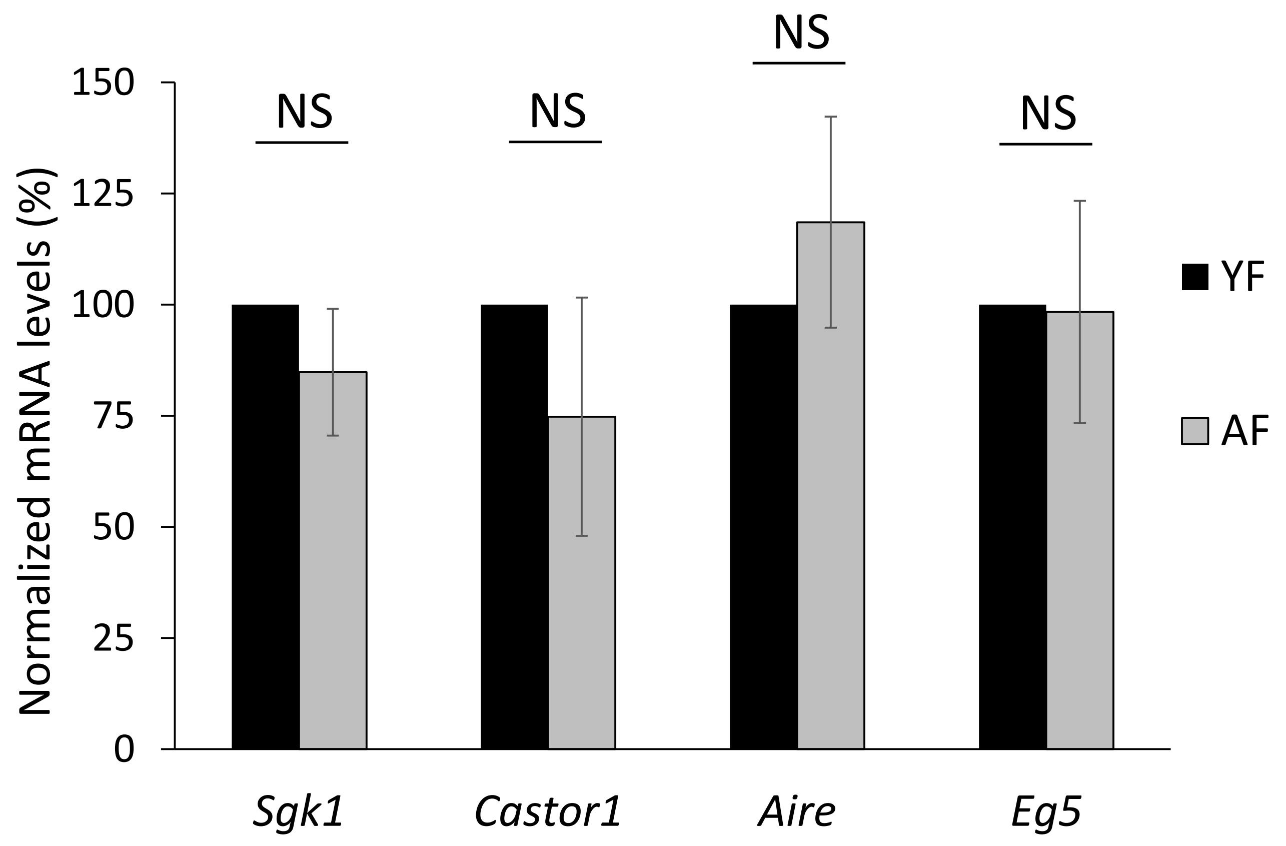

Supplementary Fig. 4

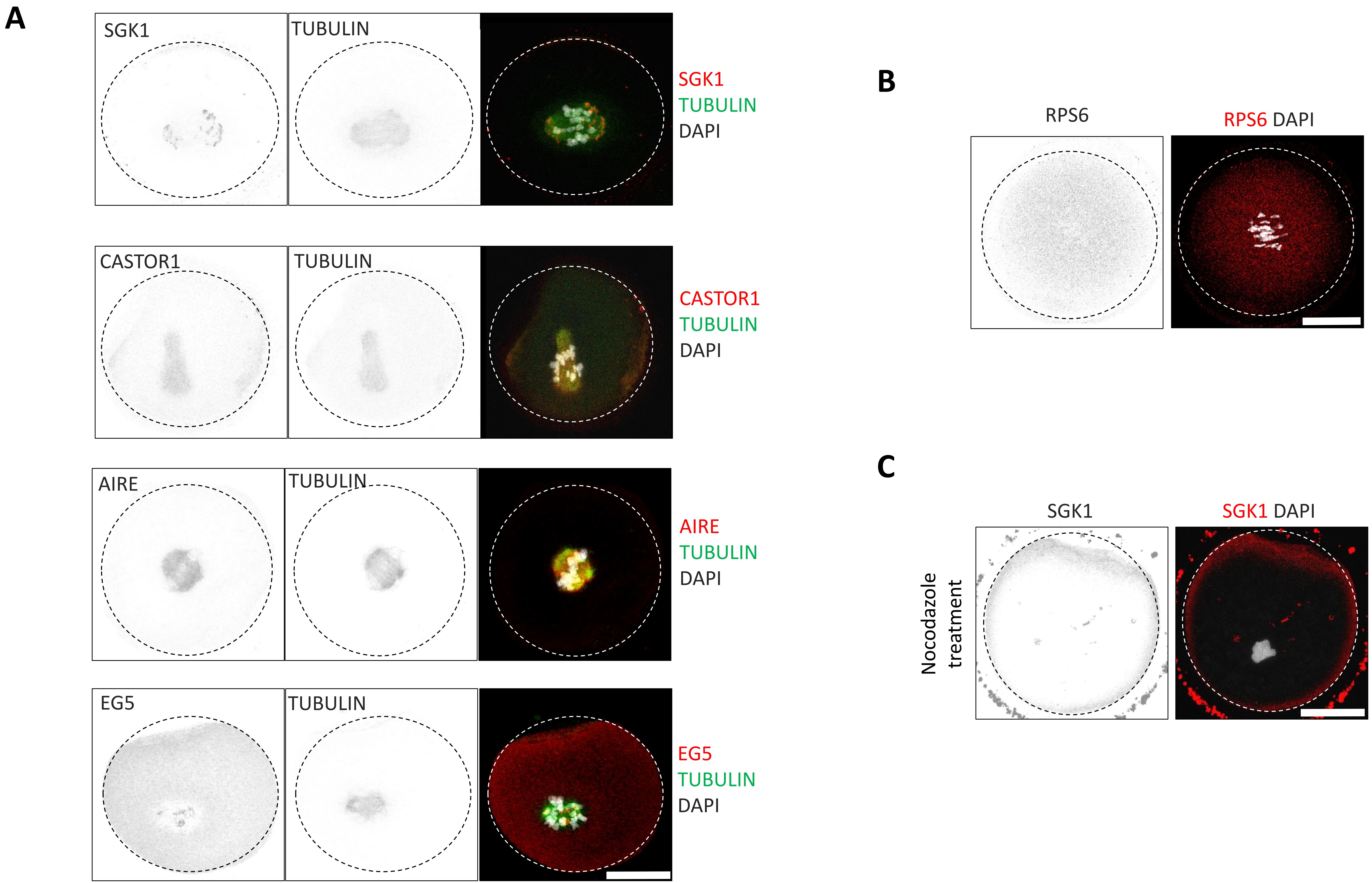

Supplementary Fig. 5

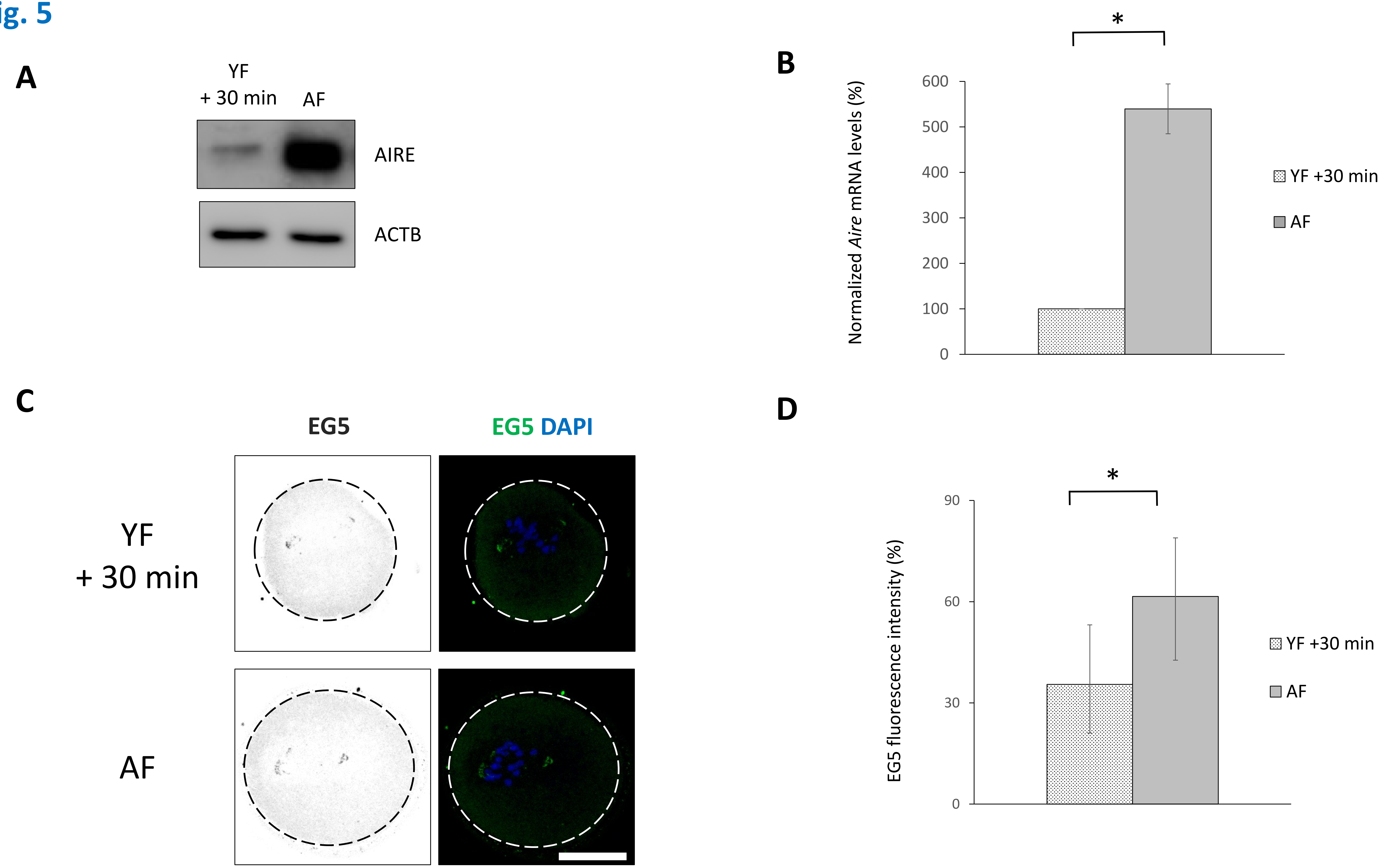

Supplementary Fig. 6

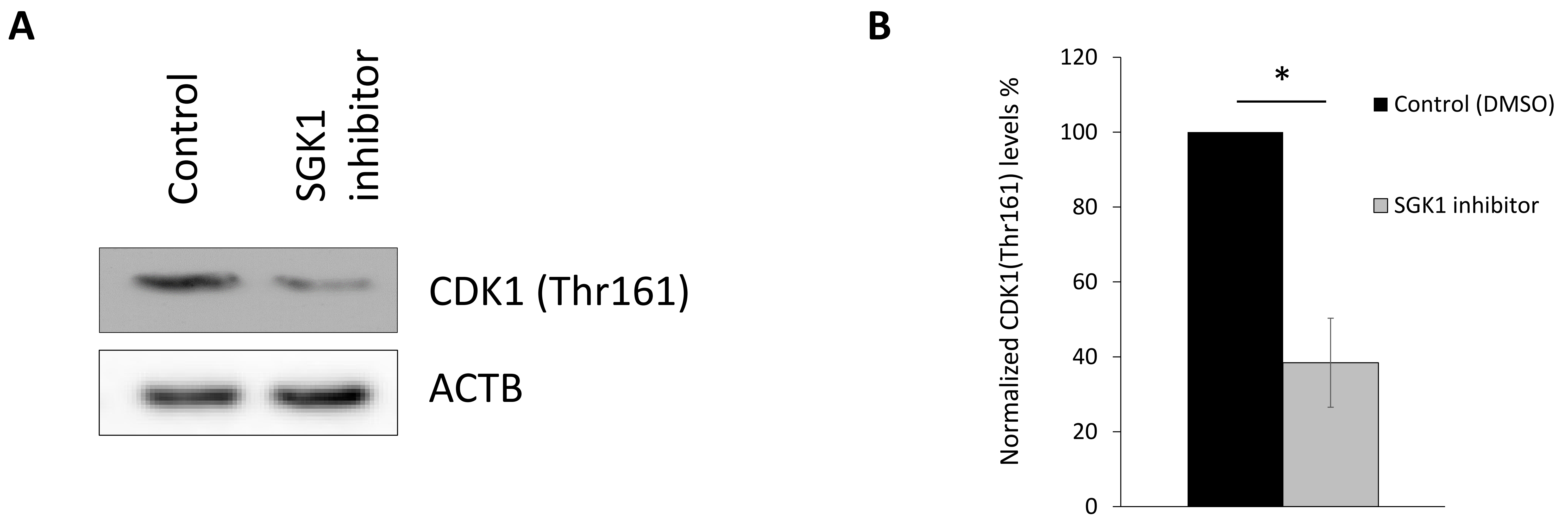

Supplementary Fig. 7

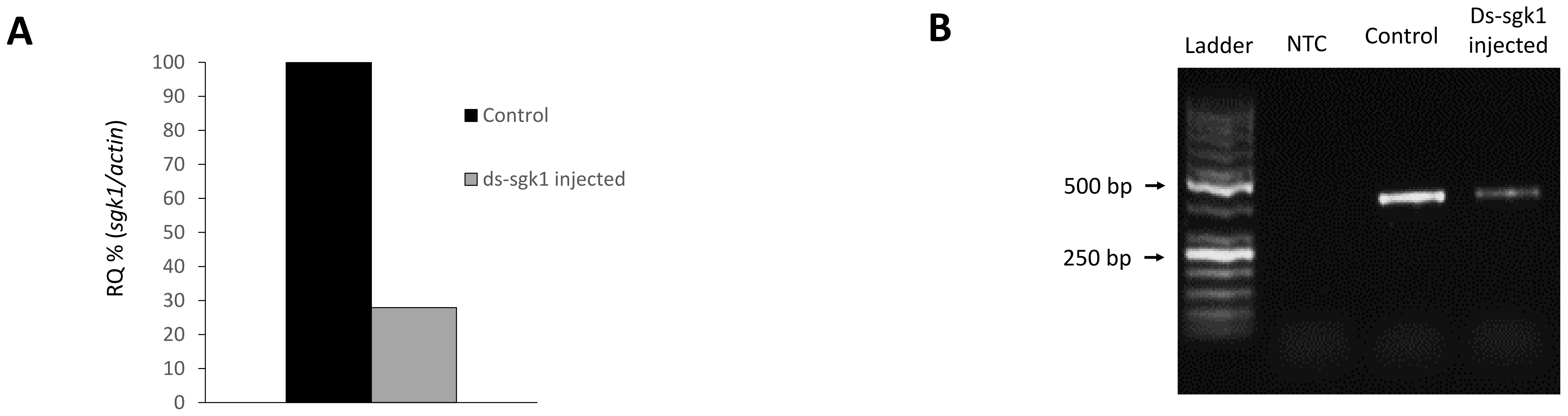

Supplementary Fig. 8

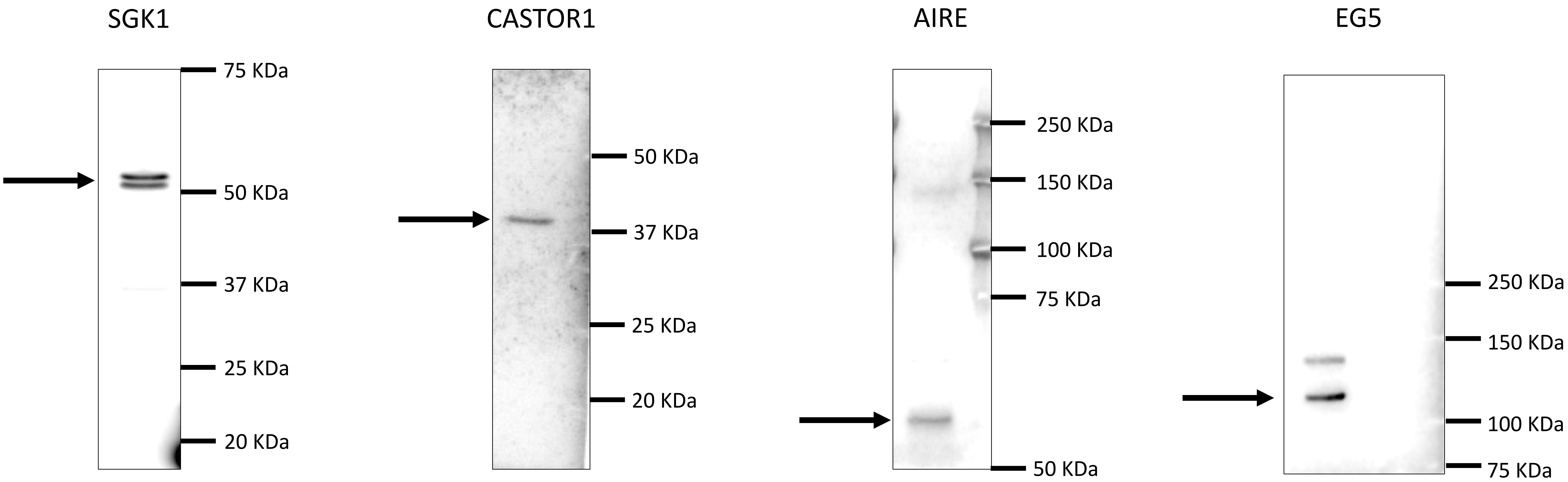

Supplementary Table 1. List of genes up/down regulated in AF compared to YF according to FC>5, FC>10 and FC>10 plus $P<0.1$.

Supplementary Table 2. List of genes in each Gene Ontology categories as seen in Fig. 1C.

Supplementary Table 3. List of genes from cluster 1,3,6,7 (increasing presence from YF-NP to YF-P, and vice versa in AF) and clusters 2,4,5 (increasing presence from increasing presence from AF-NP to AF-P, and vice versa in YF)(see Fig. S1).

Supplementary Table 4. Table of primers used for PCR (A) and (B) list of antibodies used for the study.

A

Primers		
Name	sequence	Accession number
18S-Forward	5'-CGCTCCACCAACTAAGAACG-3'	NR_003278
18S-Reverse	5'-CTCAACACGGGAAACCTCAC-3'	
28S-Forward	5'-CTAAATACCGGCACGAGACC-3'	NR_003279
28S-Reverse	5'-TTCACGCCCTCTTGAACTCT-3'	
Sgk1-Forward	5'-GGTGCCAAGGATGACTTTATGG-3'	NM_001161845, NM_001161847, NM_001161848, NM_001161849, NM_001161850, NM_011361
Sgk1-Reverse	5'-GGATCGAAGTGCCGAAGGTC-3'	
Castor1-Forward	5'-AGTAACCAAGATTGCCCGGT-3'	NM_028022
Castor1-Reverse	5'-CCCGTACCAGGATGAAGTCTG-3'	
Aire-Forward	5'-ACTCTGCTAGTCACGACCCT-3'	NM_009646, NM_001271549, NM_001271551, NM_001271550, NM_001271552, NM_001271553, NM_001271554, NM_001271555, NM_001271556, NM_001271557, NM_001271558, NM_001271559, NR_073358
Aire-Reverse	5'-CTGCAGGATGCCGTCAAATG-3'	
Eg5-Forward	5'-ATGTGTCCGCTCGTGTTTCT-3'	NM_010615
Eg5-Reverse	5'-GGTCGGTCACAAGTTCATCAATC-3'	
dsSgk1- Forward	5'- CTCATTCCAGACCGCTGACA- 3'	NM_001161845, NM_001161847, NM_001161848, NM_001161849, NM_001161850, NM_011361
dsSgk1- Reverse	5'- TTCCGGCTATAAAACGGGGG- 3'	

B

Antibodies		
Name and cat.#	Company	Experiment
SGK1 (H-4) - 28338	Santa Cruz Biotechnology	ICC
SGK1 - PA5-87746	Thermo Fisher Scientific	WB
GATSL3 (CASTOR1) (A-6) - 377114	Santa Cruz Biotechnology	ICC, WB
Aire 1 (C-2) - 373703	Santa Cruz Biotechnology	WB
AIRE Monoclonal Antibody (5H12), 14 5934 82	Thermo Fisher Scientific	ICC
EG5 (A-2) - 365593	Santa Cruz Biotechnology	ICC, WB
β Tubulin (9F3) - 2128	Cell Signalling Technology	ICC
HA-Tag(C29F4) - 3724	Cell Signalling Technology	ICC, WB
β Actin - 0061R	Biosusa	WB
Phospho-CDK1 (Thr161) - 9114	Cell Signalling Technology	WB
Ribosomal Protein S6 – 74459	Santa Cruz Biotechnology	ICC
CREST – HCT-0100	Immunovision	ICC
